# An MR-Neuroimaging Study of Structural and Vascular Brain Networks in Elderly Adults with Obesity and Diabetes who Practice Structural Yoga

**DOI:** 10.12688/f1000research.181680.1

**Published:** 2026-05-28

**Authors:** Poovitha Shruthi Paramashiva, G. Arun Maiya, Rajagopal Kadavigere, Winniecia Dkhar, Suresh Sukumar

**Affiliations:** 1 Division of Yoga, Centre for Integrative Medicine and Research, Manipal Academy of Higher Education, Manipal, Karnataka, 576104, India; 2 Department of Physiotherapy, Manipal College of Health Professions, Manipal Academy of Higher Education, Manipal, Karnataka, 576104, India; 3 Department of Radiodiagnosis and Imaging, Kasturba Medical College, Manipal Academy of Higher Education, Manipal, Karnataka, 576104, India; 4 Department of Medical Imaging Technology, Manipal College of Health Professions, Manipal Academy of Higher Education, Manipal, Karnataka, 576104, India

**Keywords:** MR neuroimaging, type 2 diabetes mellitus, obesity, yoga, brain morphometry, diffusion tensor imaging, resting-state fMRI, and elderly adults.

## Abstract

**Background:**

Obesity and type 2 diabetes mellitus (T2DM) are associated with accelerated brain atrophy, white matter microstructural disruption, and resting-state functional network dysconnectivity in elderly adults, driven by converging vascular, neuroinflammatory, and insulin-resistance mechanisms. Yoga is a recognized mind-body intervention with documented benefits for glycaemic control, vascular health, and neurocognitive function; however, no study has yet employed multimodal MR neuroimaging to systematically characterize these brain alterations in an elderly obese-diabetic population or to evaluate yoga-induced neuroplasticity within this cohort.

**Methods:**

This three-phase, prospective protocol will be conducted at Kasturba Hospital and the Center for Integrative Medicine and Research, Manipal Academy of Higher Education, Manipal, India. Phase 1 is a case-control study (n = 80; 40 obese-diabetic, 40 obese-non-diabetic; age 60–80 years) employing carotid Doppler ultrasonography, T1-weighted voxel-based morphometry and region-of-interest segmentation (SPM12/CAT12/AAL3), diffusion tensor imaging with ROI-based tractography (ExploreDTI), and resting-state fMRI (CONN toolbox), alongside the Eriksen Flanker and N-Back cognitive tasks. Phase 2 involves the systematic development and expert content-validation of a structured, AYUSH-compliant yoga module tailored for elderly obese-diabetic adults. Phase 3 applies the validated module in a 6-month pre-post intervention, with objective adherence monitoring via triaxial accelerometry and repeat of the full Phase 1 neuroimaging and cognitive battery.

**Discussion:**

This protocol addresses a critical gap in the yoga-neuroimaging literature by providing a multimodal, multi-phase framework to characterize neurovascular disease burden and evaluate structured yoga as a neurobiologically informed lifestyle intervention in a high-risk elderly population. Findings will inform AYUSH clinical guidelines, geriatric NCD prevention programs, and future randomized controlled trials.

## 1. Introduction

The global burden of non-communicable diseases (NCDs) has reached unprecedented levels, with obesity and type 2 diabetes mellitus (T2DM) constituting two of the most consequential metabolic disorders of the twenty-first century. According to the World Health Organization, the worldwide prevalence of obesity has nearly doubled since 1990, with more than 890 million adults classified as obese in 2022. ^1^ Concurrently, the International Diabetes Federation estimates that approximately 537 million adults were living with diabetes in 2021, a figure projected to rise to 783 million by 2045. ^2^ These epidemiological trends are particularly alarming in the elderly population, among whom both conditions frequently co-occur and exert synergistic deleterious effects on systemic health. In India, rapid urbanization, sedentary lifestyles, and dietary transitions have accelerated the prevalence of these metabolic comorbidities, making them priority targets under the National Program for Prevention and Control of Cancer, Diabetes, Cardiovascular Diseases, and Stroke. ^3^

Beyond their well-established cardiovascular and metabolic sequelae, obesity and T2DM exert profound adverse effects on brain structure and function. Structural neuroimaging studies have consistently demonstrated accelerated brain atrophy, reduced grey matter volume, and compromised white matter integrity in individuals with obesity and T2DM relative to metabolically healthy controls. ^4, 5^ Particularly vulnerable regions include the hippocampus, prefrontal cortex, and parietal association areas, structures critically involved in memory consolidation, executive function, and visuospatial processing. ^6^ Diffusion tensor imaging (DTI) studies further reveal microstructural white matter disruption, evidenced by reduced fractional anisotropy (FA) and elevated mean diffusivity (MD) in major fiber tracts, consistent with demyelination and axonal loss. ^7^ These neuroanatomical alterations are accompanied by functional connectivity changes detectable on resting-state functional MRI, pointing to disrupted default mode and frontoparietal networks in metabolically compromised individuals. ^8^ Taken together, this body of evidence positions obesity associated T2DM as a significant and modifiable risk factor for accelerated neurocognitive aging.

The mechanisms underlying brain alterations in obese-diabetic individuals are multifactorial, but vascular dysfunction plays a central and unifying role. Chronic hyperglycemia and dyslipidemia promote endothelial dysfunction, arterial stiffness, and impaired cerebrovascular autoregulation, collectively reducing cerebral perfusion and predisposing the brain to ischaemic injury. ^9^ Carotid artery intima-media thickness (cIMT), a validated surrogate marker of subclinical atherosclerosis, is significantly elevated in individuals with T2DM and is independently associated with reduced hippocampal volume and cognitive decline. ^10^ Furthermore, adipose tissue-derived proinflammatory cytokines, including interleukin-6 (IL-6) and tumor necrosis factor-alpha (TNF-α), disrupt the blood-brain barrier, foster neuroinflammation, and impair insulin signaling within the central nervous system, thereby compounding structural brain damage. ^11^ Insulin resistance in the brain has been specifically linked to tau hyperphosphorylation and amyloid-beta accumulation, suggesting a mechanistic overlap between T2DM and Alzheimer’s disease pathology. ^12^ Understanding these vascular-neural interactions is therefore critical to identifying therapeutic windows for intervention in elderly individuals with metabolic comorbidities.

Cognitive impairment is an important but frequently underrecognized complication of combined obesity and T2DM in the elderly. Longitudinal cohort studies indicate that individuals with both conditions face a substantially elevated risk of developing mild cognitive impairment (MCI) and dementia compared to those with either condition alone, suggesting additive or synergistic pathophysiology. ^13^ Domains most consistently affected include episodic memory, processing speed, executive function, and attentional control, all of which have significant implications for functional independence, quality of life, and adherence to self-management behaviors in this population. ^14^ Neuropsychological assessments such as the N-Back memory task and the Eriksen Flanker test provide sensitive and ecologically valid measures of working memory capacity and attentional inhibition, respectively, and have been employed in neuroimaging paradigms to establish structure-function relationships in metabolically at-risk cohorts. ^15^ The use of such cognitive probes in conjunction with multimodal MRI, therefore, offers a comprehensive framework for characterizing neurocognitive vulnerability in elderly adults with obesity and T2DM.

Yoga, a traditional Indian mind-body practice encompassing physical postures (asanas), breath regulation (pranayama), and meditative techniques, has attracted growing scientific interest as a holistic lifestyle intervention for metabolic and neurological health. A robust evidence base now supports the efficacy of yoga in improving glycaemic control, reducing body mass index (BMI), lowering systolic blood pressure, and attenuating inflammatory biomarkers in individuals with T2DM and obesity. ^16, 17^ Yoga has been shown to enhance vagal tone and modulate the hypothalamic-pituitary-adrenal (HPA) axis, thereby reducing allostatic load and counteracting the sympathoadrenal overactivation characteristic of metabolic syndrome. ^18^ Regarding brain health, preliminary neuroimaging studies have reported that yoga is associated with increases in cortical thickness, hippocampal volume, and grey matter density in regular practitioners compared to non-practitioners, accompanied by improvements in cognitive performance and emotional regulation. ^19, 20^ These structural adaptations are thought to be mediated in part by yoga-induced elevations in brain-derived neurotrophic factor (BDNF) and insulin-like growth factor-1 (IGF-1), both of which promote neuroplasticity and neurogenesis in the hippocampal dentate gyrus. ^21^

Despite these promising findings, significant gaps persist in the literature. First, most yoga intervention studies have been conducted in younger or otherwise healthy populations, with limited generalization to elderly adults burdened with metabolic comorbidities. ^22^ Second, existing neuroimaging investigations of yoga have largely relied on cross-sectional designs, precluding causal inference regarding the direction and magnitude of yoga-induced brain changes. ^23^ Third, to date, no studies have employed a multi-sequence MRI protocol combining volumetric morphometry, DTI, and resting-state fMRI to concurrently characterize structural, microstructural, and functional brain network adaptations to yoga in elderly obese-diabetic individuals. Fourth, there remains a lack of culturally validated, evidence-based, structured yoga modules specifically tailored to the physiological constraints and comorbidity profiles of this demographic, constituting a critical barrier to clinical translation. ^24^ Addressing these gaps requires a rigorous, prospective, and multimodal investigation.

## 2. Protocol

### 2.1 Study design

This study is a three-phase, prospective investigation integrating a cross-sectional case-control design (Phase 1), an instrument development and validation phase (Phase 2), and a pre-post interventional design (Phase 3). The protocol was approved by the Indian Council of Medical Research (ICMR) under the Call for Investigator-Initiated Research Proposals for Small Extramural Grants (Priority Area: NCD Risk Factors — Diabetes; Aging and Elderly Health).

### 2.2 Study setting

All neuroimaging data (MRI and Doppler) will be acquired at the Department of Radiodiagnosis and Imaging, Kasturba Hospital, Manipal, using a 3-Tesla United Imaging uMR 780 MRI system. Cognitive assessments will be conducted in a dedicated, distraction-free testing room in the same department. The yoga module development and validation (Phase 2) will be carried out at the Division of Yoga, Centre for Integrative Medicine and Research (CIMR), Manipal. Physical activity data will be captured using accelerometers.

### 2.3 Phase 1

**2.3.1 Objectives**

The primary objective of Phase 1 is to determine and compare vascular, structural, and functional brain changes, along with alterations in cognitive function, between obese elderly adults with T2DM and those without T2DM using multimodal MR neuroimaging.

**2.3.2 Study design**

Case-control study.

**2.3.3 Inclusion criteria**

• Age 60–80 years, both sexes.
• Clinical diagnosis of type 2 diabetes mellitus for the obese-diabetic group.
• Body mass index (BMI) ≥ 25 kg/m ^2^ as per Asia-Pacific guidelines for obesity classification. ^25^
• Able to provide written informed consent independently or through a legally authorized representative.

**2.3.4 Exclusion criteria**

• Presence of neurological or psychiatric conditions known to affect brain morphology or cognitive function (e.g., Parkinson’s disease, major depressive disorder, schizophrenia, prior stroke or traumatic brain injury).
• History of neurodegenerative disease (e.g., Alzheimer’s disease, vascular dementia).
• Type 1 diabetes mellitus or secondary forms of diabetes.
• Active malignancy or any systemic illness likely to confound neuroimaging or cognitive assessments.
• Contraindications to MRI (metallic implants, claustrophobia, cardiac pacemakers).
• Current or recent (within 6 months) regular yoga practice (≥ 2 sessions per week).
• Severe hearing or visual impairment precluding cognitive testing.

**2.3.5 Sample size**

Sample size was estimated using G*Power 3.1 software. A multivariate analysis of variance (MANOVA) power analysis was performed with an assumed medium effect size (f ^2^ = 0.25), a significance level of α = 0.05, and a desired statistical power of 0.80. This yielded a minimum total sample of 80 participants. Accounting for anticipated attrition and protocol deviations, 40 participants will be recruited to each group (obese-diabetic and obese-non-diabetic), yielding a total of 80 participants for Phase 1.

**2.3.6 Baseline assessments**

**2.3.6.1 Physical activity assessment**

Physical activity will be objectively measured using a triaxial accelerometer worn on the participant’s dominant thigh for 7 consecutive days, including at least 2 weekend days, in accordance with published guidelines for accelerometry in older adults. ^26^ The device will record total activity counts and time spent in sedentary, light, moderate, and vigorous-intensity physical activity, expressed as metabolic equivalent of task (MET) minutes per week. In addition, the validated Global Physical Activity Questionnaire (GPAQ) will be administered by a trained research assistant to capture self-reported physical activity across three domains (work, transport, and leisure) and sedentary time. ^27^ The GPAQ requires approximately 5–10 minutes to complete and will be administered after detailed verbal and written instructions are provided.

**2.3.6.2 Anthropometric and metabolic measurements**

Height will be measured to the nearest 0.1 cm using a calibrated stadiometer, and body weight will be recorded to the nearest 0.1 kg using a digital weighing scale, with participants barefoot and in light clothing. BMI will be calculated as weight (kg) divided by height squared (m ^2^). Waist circumference will be measured at the midpoint between the lowest rib and the iliac crest. Fasting blood glucose and HbA1c will be recorded from participants’ clinical laboratory reports.

**2.3.6.3 Vascular assessment: carotid doppler ultrasonography**

Carotid Doppler ultrasonography will be performed with participants seated following a 10-minute rest period. A 7–15 MHz multifrequency linear array transducer will be used with a high-resolution B-mode ultrasound machine. Peak systolic velocity (PSV), end-diastolic velocity (EDV), and resistive index (RI) of the common carotid artery (CCA) will be recorded bilaterally. Carotid artery diameter will be measured at the far wall of the CCA, 1 cm proximal to the carotid bifurcation. Endothelial shear stress will be calculated from the velocity and diameter values. All measurements will be performed by the same trained sonographer throughout the study to minimize inter-rater variability. A minimum of three consecutive cardiac cycles will be averaged for each measurement.

**2.3.6.4 Neuroimaging protocol**

All MRI data will be acquired using the 3-Tesla United Imaging uMR 780 scanner at the Department of Radiodiagnosis and Imaging, Kasturba Hospital, Manipal. Participants will be positioned supine with their head immobilized using foam padding to minimize motion artifacts. A 32-channel head coil will be used for all sequences. The imaging protocol comprises four sequences as follows.

**Sequence 1: Structural MRI — Volumetric brain morphometry**

A T1-weighted three-dimensional Fast Spoiled Gradient Echo (FSPGR) sequence will be acquired for voxel-based morphometry (VBM) analysis. Imaging parameters will include repetition time (TR) = 7.8 ms; echo time (TE) = 3.1 ms; inversion time (TI) = 1010 ms; flip angle = 10°; field of view (FOV) = 256 × 256 mm; voxel size = 1 × 1 × 1 mm isotropic; 176 contiguous axial slices. Post-processing and analysis will be performed in MATLAB (MathWorks) using the SPM12 toolbox (Wellcome Centre for Human Neuroimaging, UCL) with the CAT12 (Computational Anatomy Toolbox, version 12) extension. ^28^ The standard CAT12 cross-sectional pipeline will be applied, comprising: (1) bias-field correction and denoising; (2) unified segmentation into grey matter (GM), white matter (WM), and cerebrospinal fluid (CSF) tissue classes; (3) diffeomorphic registration to the MNI152 standard space using the Geodesic Shooting algorithm (DARTEL); and (4) modulation of segmented images to preserve absolute tissue volumes following normalisation. For ROI-based segmentation, the CAT12 automated parcellation will be applied using the Automated Anatomical Labeling (AAL3) atlas, extracting absolute and relative grey matter volumes for bilateral regions of interest, including the hippocampus, entorhinal cortex, prefrontal cortex, anterior cingulate cortex, insula, and parietal association cortex. Total intracranial volume (TIV) will be extracted from the Jacobian determinant maps and used as a covariate in all volumetric analyses to account for individual differences in head size.

**Sequence 2: Diffusion tensor imaging (DTI)**

White matter microstructural integrity will be assessed using a single-shot echo-planar DTI sequence acquired with participants in the supine position. Acquisition parameters will include TR = 4237 ms; TE = 87.3 ms; b-values = 0 and 1000 s/mm ^2^; 32 non-collinear diffusion gradient directions; FOV = 220 × 220 mm; voxel size = 1.72 × 1.72 × 2 mm; 45 axial slices; and GRAPPA acceleration factor = 2. DTI data will be pre-processed and analyzed using ExploreDTI. ^29^ Pre-processing will include: (1) signal drift correction; (2) Gibbs ringing suppression; (3) eddy current and subject motion correction using a robust tensor estimation approach with outlier rejection (REKINDLE algorithm); and (4) brain extraction. The diffusion tensor will be estimated at each voxel using a non-linear least squares fitting procedure. ROI-based tractography will be performed using deterministic streamline tractography (Euler integration, step size = 1 mm, FA threshold = 0.20, maximum turning angle = 30°). White matter tracts of primary interest — including the corpus callosum, corticospinal tract, superior longitudinal fasciculus, inferior fronto-occipital fasciculus, cingulum, and uncinate fasciculus — will be reconstructed using atlas-based ROI placement guided by the JHU white matter atlas. Mean values of fractional anisotropy (FA), mean diffusivity (MD), radial diffusivity (RD), and axial diffusivity (AD) will be extracted per tract per participant for between-group and pre-post comparisons.

**Sequence 3: Resting-State functional MRI (rs-fMRI)**

Resting-state BOLD (Blood Oxygenation Level-Dependent) fMRI will be acquired using a gradient-echo echo-planar imaging (EPI) sequence. Parameters: TR = 3000 ms; TE = 30 ms; flip angle = 80°; FOV = 230 × 230 mm; voxel size = 3.5 × 3.5 × 3.5 mm; 40 interleaved axial slices. Participants will be instructed to lie still with eyes open, fixated on a cross projected on a screen, and to refrain from deliberate mental activity. RS-fMRI data will be preprocessed using the CONN toolbox (v22.a) in MATLAB, including slice-timing correction, realignment, co-registration to T1 structural image, normalization to MNI space, and spatial smoothing (6 mm FWHM Gaussian kernel). ^30^ Independent component analysis (ICA) will be used to identify and remove noise components (motion, physiological artifacts). Default mode network (DMN), frontoparietal network (FPN), and salience network (SN) connectivity will be examined as primary functional outcomes using seed-based and ICA-based approaches.

**2.3.6.5 Cognitive assessment**

**Task 1: Eriksen flanker task**

The Eriksen Flanker task will be used to assess selective attention and response inhibition. ^15^ Participants will be seated approximately 60 cm from a 24-inch monitor, with feet flat on the floor. A central target letter flanked by congruent or incongruent distractor letters will be presented for 1000 ms, followed by a 500 ms inter-stimulus interval. Participants will be instructed to respond to the central target letter while ignoring flanking stimuli, pressing ‘Q’ for target letters ‘H’ or ‘K’ and ‘P’ for target letters ‘S’ or ‘C’. A total of 96 trials (48 congruent, 48 incongruent) will be administered following 12 practice trials. Outcome measures will include mean reaction time (ms) and response accuracy (%) for congruent and incongruent conditions, and the flanker interference effect (incongruent minus congruent reaction time).

**Task 2: N-Back memory task**

Working memory capacity and cognitive processing speed will be assessed using the N-Back paradigm. ^31^ Letter stimuli will be presented sequentially on screen at a rate of one per 2000 ms. Four conditions will be administered in sequence: 0-Back (respond when the letter ‘M’ appears), 1-Back, 2-Back, and 3-Back (respond when the current letter matches the letter presented 1, 2, or 3 positions previously, respectively). Each level will comprise 30 trials (10 targets, 20 lures/non-targets). Response accuracy (d-prime signal detection metric) and median reaction time will serve as the primary cognitive outcome measures for each N-Back level.

**2.3.6.6 Outcome measures**

**Primary outcomes**

• Carotid artery and superficial femoral artery diameter, PSV, EDV, RI, and endothelial shear stress (Doppler ultrasonography).
• Regional grey matter volume and TIV (T1-weighted VBM).
• FA, MD, RD, and AD of major white matter tracts (DTI).
• Resting-state functional connectivity of the DMN, FPN, and SN (rs-fMRI).

**Secondary outcomes**

• Eriksen Flanker task: mean reaction time and accuracy for congruent and incongruent conditions; flanker interference effect.
• N-Back task: d-prime and median reaction time across 0-Back, 1-Back, 2-Back, and 3-Back conditions.

**2.3.6.7 Statistical analysis**

All statistical analyses will be performed using Jamovi. Demographic and clinical characteristics will be summarised using descriptive statistics: mean ± standard deviation (SD) for normally distributed continuous variables; median and interquartile range (IQR) for skewed data; and frequencies with percentages for categorical variables. Normality will be assessed using the Shapiro-Wilk test. Group differences in neuroimaging and cognitive outcomes will be examined using independent-samples t-tests or Mann-Whitney U tests, as appropriate. A two-way ANOVA will be used to investigate interactions between group and sex or age; a nonparametric alternative (Kruskal-Walli’s test with Dunn’s post hoc correction) will be applied when distributional assumptions are violated. Associations between neuroimaging metrics, vascular parameters, metabolic indices, and cognitive performance will be explored using Pearson’s or Spearman’s correlation coefficients and multiple linear regression models, adjusting for age, sex, years of education, and physical activity level. A significance threshold of p < 0.05 (two-tailed) will be applied throughout.

### 2.4 Phase 2

**2.4.1 Objective**

The objective of Phase 2 is to develop and content-validate a structured yoga module tailored to the physiological capabilities, safety requirements, and therapeutic needs of elderly adults with comorbid obesity and T2DM.

**2.4.2 Module development**

The yoga module will be developed through a systematic three-phase approach aligned with the Ministry of AYUSH, Government of India, guidelines.

**Step 1: Literature review**

A comprehensive review of traditional yogic texts including the Hatha Yoga Pradipika, ^32^ Gheranda Samhita, ^33^ Light on Yoga, ^34^ and Asana Pranayama Mudra Bandha ^35^ will be conducted alongside a structured search of contemporary scientific databases (PubMed, Scopus, Web of Science, Embase, and Ovid) using the key terms: yoga, elderly, type 2 diabetes, obesity, vascular health, brain, cognitive function, and neuroimaging. Studies reporting specific yoga practices with documented benefits for glycaemic control, vascular function, brain structure, or cognitive outcomes will be extracted and synthesized.

**Step 2: Integration and selection of practices**

Yoga practices identified as having therapeutic potential for vascular health, brain structural and functional improvement, and cognitive enhancement will be collated. These will be evaluated for their safety and practicability in the context of the target population’s physical limitations, comorbidities (osteoarthritis, reduced balance, cardiorespiratory deconditioning), and age-related contraindications to inversions, deep twists, and high-load postures. Selected practices will encompass asanas (physical postures), pranayama (breath regulation), and dharana/dhyana (mindfulness and concentration techniques).

**Step 3: Adaptation and manual preparation**

Selected practices will be adapted to accommodate the functional capacity of elderly obese-diabetic individuals. Modifications will include the use of props (chairs, bolsters, straps), reduced range-of-motion variants, seated and supine alternatives to standing postures, and graded progressions. A structured yoga manual will be produced, containing illustrated instructions, duration and frequency specifications, contraindication notices, and safety guidelines. The manual will be bilingual (English and Kannada) to ensure accessibility in the local population.

**2.4.3 Expert validation**

The completed yoga module will be submitted to a panel of eleven certified expert validators with recognized expertise in geriatric yoga, diabetes management, or integrative medicine. Validators will include yoga therapists, physiotherapists specializing in geriatric rehabilitation, endocrinologists, and exercise physiologists. Each expert will receive the module, along with a structured validation form and a written explanation of the study’s purpose and the validation process. Signed informed consent will be obtained prior to validation.

The validation form will assess each practice across three dimensions: usefulness, appropriateness, and relevance, rated on a 4-point Likert scale. The Content Validity Ratio (CVR) for each item will be calculated using Lawshe’s formula ^36^:

$$\mathrm{CVR}=\left(n_{e}-N/2\right)/\left(N/2\right)$$

where n _e_ is the number of experts rating the item as ‘essential,’ and N is the total number of experts. Based on Lawshe’s table for 11 experts, a minimum CVR of 0.59 is required for an item to be retained (corresponding to agreement by ≥9 out of 10 experts) [39]. Items receiving agreement from fewer than 80% of experts will be reviewed, modified, or excluded based on expert commentary. The Content Validity Index (CVI) for the overall module will be computed as the mean CVR across all retained items.

**2.4.4 Outcomes**

The primary output of Phase 2 will be a content-validated, AYUSH-compliant, structured yoga module, accompanied by a bilingual participant manual, a facilitator guide, and a standardized session-delivery checklist. The validated module will serve as the basis for the Phase 3 intervention.

### 2.5 Phase 3

**2.5.1 Objective**

Phase 3 aims to investigate the effects of the validated structured yoga module on vascular health, structural and functional brain networks, and cognitive function in elderly adults with comorbid obesity and T2DM, using multimodal MR neuroimaging before and after a 6-month intervention period.

**2.5.2 Study design**

Pre-test post-test interventional study with repeated multimodal neuroimaging and cognitive assessment.

**2.5.3 Participants**

Participants recruited in Phase 1 will be eligible for Phase 3. Inclusion and exclusion criteria will be reconfirmed at the time of enrolment into Phase 3. Participants who have developed new contraindications to yoga or MRI during the Phase 1 to Phase 3 interval will be excluded.

**2.5.4 Intervention: delivery**

The structured yoga module validated in Phase 2 will be delivered to each participant as a printed bilingual manual accompanied by an illustrative video guide. An initial in-person orientation session (approximately 60 minutes for the first 15 days) will be conducted at CIMR, Manipal, during which a certified yoga instructor will demonstrate all practices, correct technique, and address safety questions.

**2.5.5 Intervention: frequency and duration**

Participants will be asked to practice yoga for at least 5 days per week for 6 months. Each session will be approximately 45–60 minutes in duration, incorporating a warm-up asana sequence (10 minutes), core therapeutic asanas (25–30 minutes), pranayama (10 minutes), and relaxation/dharana (5–10 minutes).

**2.5.6 Physical activity monitoring**

Objective physical activity will be continuously monitored throughout the 6-month intervention using an accelerometer worn on the dominant thigh during all yoga sessions and other physical activities. Participants will be instructed to wear the accelerometer throughout waking hours (except during bathing or water activities) and to activate a dedicated event marker button at the commencement and completion of each yoga session.

**2.5.7 Remote adherence monitoring**

To support adherence and participant retention, a dedicated remote monitoring protocol will be implemented. Research staff will contact participants weekly via their preferred messaging platform (WhatsApp or SMS) to enquire about yoga practice status, address queries, and provide motivational support. Participants will also be encouraged to use commercially available mobile fitness-tracking applications (e.g., Google Fit) or wearable fitness devices (e.g., smartwatches) to self-monitor daily physical activity. Participants will maintain session logs in a structured diary provided at enrolment.

**2.5.8 Post-Intervention assessment**

Following the 6-month intervention period, all participants will undergo a complete repeat of the neuroimaging protocol and cognitive assessments as described in Phase 1. Physical activity, anthropometric, and metabolic measurements will also be repeated using identical procedures and equipment.

**2.5.9 Outcome measures**

**Primary outcomes**

• Change from baseline to 6 months in carotid artery hemodynamic parameters.
• Change from baseline in regional grey matter volume and TIV.
• Change from baseline in DTI metrics of major white matter tracts.
• Change from baseline in resting-state functional connectivity of the DMN, FPN, and SN (rs-fMRI).

**Secondary outcomes**

• Change from baseline in the Eriksen Flanker task performance (reaction time and accuracy).
• Change from baseline in N-Back task performance (d-prime and reaction time across all levels).

**2.5.10 Statistical analysis**

Pre- and post-intervention neuroimaging and cognitive outcomes will be compared using paired samples t-tests (or Wilcoxon signed-rank tests for non-normally distributed data). Mixed-effects linear regression models will be used to examine the longitudinal trajectory of outcome variables, adjusting for relevant covariates (age, sex, baseline physical activity, HbA1c, and medication use). The association between intervention-induced changes in neuroimaging metrics and changes in vascular, metabolic, and cognitive outcomes will be examined using partial correlations and multivariate regression. Effect sizes (Cohen’s d and partial η ^2^) will be reported for all primary outcomes.

## 3 Dissemination

The findings of this study will be disseminated through multiple channels targeting both scientific and public health audiences. Primary results from each phase will be submitted for publication in peer-reviewed, open-access journals indexed in Scopus, with preference for journals specializing in neuroimaging, integrative medicine, and geriatric health.

Conference presentations will be submitted to national and international meetings in the fields of neuroscience, yoga and integrative medicine, and diabetes research. Research summaries will be shared with the Indian Council of Medical Research through mandatory progress and final technical reports as stipulated under the ICMR Small Extramural Grant conditions.

## 4 Study status

The study has received approval from the Indian Council of Medical Research (ICMR) under the Small Extramural Grants scheme (Priority Area: NCD Risk Factors — Diabetes; Aging and Elderly Health). Approval from the Institutional Ethics Committee (IEC) of Kasturba Hospital, Manipal, was sought. Prospective registration with the Clinical Trials Registry — India (CTRI) is completed. Concurrent activities include finalizing the research team, procuring study equipment (accelerometers and an ultrasound machine), and preparing participant-facing study materials.

## 5 Discussion

The current protocol describes a comprehensive, three-phase investigation designed to characterize alterations in the vascular, structural, and functional brain networks associated with obesity and T2DM in elderly adults, and to evaluate the neuromodulatory potential of a validated, structured yoga intervention using multimodal MR neuroimaging. To our knowledge, this is the first study to employ a concurrent multi-sequence MRI protocol, encompassing T1-weighted VBM with CAT12 ROI segmentation, ExploreDTI-based white matter tractography, resting-state fMRI, and carotid Doppler ultrasonography, specifically in an elderly obese-diabetic population undergoing a structured yoga intervention.

The decision to adopt a three-phase sequential design reflects the complexity of the research question and the need to establish a robust methodological foundation before proceeding to the intervention. Phase 1 addresses the critical need for a detailed neurobiological characterization of the target population prior to any therapeutic application, providing both the baseline comparator data and the evidence base that informed the structure of the Phase 3 intervention. Phase 2 ensures that the yoga module delivered in Phase 3 is not only scientifically grounded but also culturally appropriate, practically feasible, and formally validated for safety and content, a step conspicuously absent in most existing yoga intervention trials, which have frequently relied on ad hoc or unstandardized practice regimens. Phase 3 then applies the validated module in a prospective pre-post design, enabling within-subject quantification of neuroplastic change across all imaging and cognitive domains characterized in Phase 1.

Several limitations of the present study design merit acknowledgment. The study population is drawn from a single tertiary hospital in coastal Karnataka, which may limit the generalisability of the findings to other regions of India and to populations with differing levels of yoga familiarity, dietary patterns, and genetic risk profiles for T2DM and obesity. Second, the 6-month intervention window, while among the longest in the yoga-neuroimaging literature, may be insufficient to detect macrostructural volumetric changes in highly atrophied regions; longer follow-up periods and multi-site designs should be incorporated in future work.

Notwithstanding these limitations, the present study addresses a genuinely underserved research priority. Elderly adults with obesity and T2DM represent one of the fastest-growing demographic segments in India and globally, yet they remain largely excluded from neuroimaging research and targeted yoga intervention trials alike. The multimodal, multi-phase design of this protocol, combining carotid vascular imaging, structural morphometry, white matter tractography, resting-state fMRI, and formal yoga module validation under a single framework, constitutes a methodologically rigorous and clinically meaningful contribution to the field. If the hypothesized neuroplastic and vascular benefits of structured yoga are confirmed, the validated module and accompanying evidence base generated by this study could be directly integrated into community-level NCD prevention programs, AYUSH clinical practice guidelines, and geriatric rehabilitation protocols across India.

## Ethical considerations

The study will be conducted in strict accordance with the Declaration of Helsinki and ICMR’s National Ethical Guidelines for Biomedical and Health Research Involving Human Participants. IEC approval was obtained from the Institutional Ethics Committee, Kasturba Hospital, Manipal (IEC1: 520/2025). All participants will receive oral and written explanations of the study purpose, procedures, risks, benefits, confidentiality safeguards, and their right to withdraw at any time without affecting their clinical care. Signed written informed consent will be obtained.

## Data Availability

No data are associated with this article. This is a study protocol; no participant data have been collected at the time of publication. This protocol has been prepared in accordance with the Standard Protocol Items: Recommendations for Interventional Trials (SPIRIT 2025) guidelines. The completed SPIRIT 2013 checklist has been deposited in the Figshare repository. Repository name: An MR-Neuroimaging Study of Structural and Vascular Brain Networks in Elderly Adults with Obesity and Diabetes who Practice Structural Yoga. https://doi.org/10.6084/m9.figshare.32151694. ^37^ License: CC0 1.0 Universal (CC0 1.0) Public Domain Dedication. Clinical Trials Registry — India (CTRI): CTRI/2025/12/098738. Registered: 10/12/2025. URL: https://ctri.nic.in/Clinicaltrials/pmaindet2.php?EncHid=MTQ4MTg5&Enc=36227.78897&userName=
