# Supplementary material for: An MR-Neuroimaging Study of Structural and Vascular Brain Networks in Elderly Adults with Obesity and Diabetes who Practice Structural Yoga
Source: F1000Res. 2026 May 28;15:830. [Version 1] doi: 10.12688/f1000research.181680.1 (PMC13287981; doi:10.12688/f1000research.181680.1)
Supplement: Peer review history

# Reviewer report: Reviewer response for version 1

**DOI:** 10.5256/f1000research.200549.r490461

**Published:** 2026-06-23

**Authors:** Magdy Ali

**Affiliations:** 1 Gulf Medical University, Ajman, Ajman, United Arab Emirates

The manuscript is scientifically valuable and methodologically strong. The study design is comprehensive and clinically relevant. However, several issues should be addressed before indexing: 

 Primary and secondary hypotheses are not explicitly stated.

 Section requiring revision:

 Introduction (Pages 4–5)

 Sample size estimation lacks justification regarding the selected primary neuroimaging endpoint and the source of the assumed effect size.

 Section requiring revision:

 Section 2.3.5. Sample Size (Page 6)

 MRI motion correction procedures for elderly participants are insufficiently detailed.

 Section requiring revision:

 Section 2.3.6.4. Neuroimaging Protocol (Pages 6–7)

 Criteria for exclusion due to excessive head motion are not reported.

 Section requiring revision:

 Section 2.3.6.4. Sequence 2 (DTI) and Sequence 3 (rs-fMRI) (Page 7)

 Potential confounding effects of medications (e.g., antidiabetic, antihypertensive, and lipid-lowering agents) are not adequately addressed.

 Section requiring revision:

 Sections 2.3.6.2. Anthropometric and Metabolic Measurements and 2.5.10. Statistical Analysis (Pages 6 and 11)

 Statistical correction methods for multiple neuroimaging comparisons are not specified.

 Section requiring revision:

 Sections 2.3.6.7. and 2.5.10. Statistical Analysis (Pages 8 and 11)

 The rs-fMRI preprocessing pipeline lacks sufficient methodological detail regarding nuisance regression, motion regression, and temporal filtering.

 Section requiring revision:

 Section 2.3.6.4. Sequence 3: Resting-State fMRI (Page 7)

 Minimum adherence criteria for the intervention analysis are not defined.

 Section requiring revision:

 Sections 2.5.6. Physical Activity Monitoring and 2.5.7. Remote Adherence Monitoring (Page 10)

 Intra-rater reliability and measurement reproducibility for carotid Doppler ultrasonography are not described.

 Section requiring revision:

 Section 2.3.6.3. Vascular Assessment (Page 6)

 The protocol does not explain how incidental neuroimaging findings will be managed.

 Section requiring revision:

 Ethical Considerations (Page 12)

 The Discussion section could be more concise and focused on the protocol's significance and limitations.

 Section requiring revision:

 Discussion (Pages 11–12)

 Several references should be updated to include more recent literature related to multimodal neuroimaging, diabetes-associated brain changes, and yoga interventions.

 Section requiring revision:

 Reference List (Pages 12–13)

 Minor formatting inconsistencies are present in MRI acquisition parameters, units, and statistical notation.

 Section requiring revision:

 Methods (Pages 6–11)

 The protocol does not clearly describe how participant attrition and missing data during the 6-month intervention will be handled statistically.

 Section requiring revision:

 Sections 2.5.5. Intervention Frequency and Duration and 2.5.10. Statistical Analysis (Pages 10–11)

 The limitation of single-center recruitment could be discussed more thoroughly with respect to the external validity of the findings.

 Section requiring revision:

 Discussion—Limitations (Page 11)

Is the study design appropriate for the research question?

Partly

Is the rationale for, and objectives of, the study clearly described?

Yes

Are sufficient details of the methods provided to allow replication by others?

Partly

Are the datasets clearly presented in a useable and accessible format?

Yes

Reviewer Expertise:

MR-Neuroimaging

I confirm that I have read this submission and believe that I have an appropriate level of expertise to confirm that it is of an acceptable scientific standard, however I have significant reservations, as outlined above.

# Reviewer report: Reviewer response for version 1

**DOI:** 10.5256/f1000research.200549.r490460

**Published:** 2026-06-22

**Authors:** Prashant Mukkannvar

**Affiliations:** 1 KLE University, Hubballi, Karnataka, India

This is a well-designed and relevant study protocol. The methodology is robust and clearly described. The multimodal neuroimaging approach is a major strength. The structured yoga intervention is appropriate and innovative. This protocol can generate valuable evidence on brain health, diabetes, obesity, and healthy aging in older adults. The study objectives are clear and clinically relevant. The outcome measures are comprehensive and well selected. The findings may support future clinical practice and research in integrative healthcare.

Is the study design appropriate for the research question?

Yes

Is the rationale for, and objectives of, the study clearly described?

Yes

Are sufficient details of the methods provided to allow replication by others?

Yes

Are the datasets clearly presented in a useable and accessible format?

Yes

Reviewer Expertise:

physical therapy, MSK pain, pain science, pain education

I confirm that I have read this submission and believe that I have an appropriate level of expertise to confirm that it is of an acceptable scientific standard.

# Reviewer report: Reviewer response for version 1

**DOI:** 10.5256/f1000research.200549.r490463

**Published:** 2026-06-11

**Authors:** Manna Debnath

**Affiliations:** 1 Department of Medical Radiology and Imaging Technology, Royal School of Medical and Allied Sciences (RSMAS), Royal Global University, Guwahati, Assam, India; 2 Charotar University of Science and Technology, Changa, Gujarat, India

The study addresses an important and contemporary topic, integrating advanced MR-neuroimaging techniques with lifestyle-based interventions in a clinically relevant population. The methodology is appropriate, the data analysis is robust, and the findings are clearly presented and well interpreted. The manuscript is well structured, scientifically sound, and contributes valuable insights to the fields of neuroimaging, aging, metabolic disorders, and complementary medicine.

Is the study design appropriate for the research question?

Yes

Is the rationale for, and objectives of, the study clearly described?

Yes

Are sufficient details of the methods provided to allow replication by others?

Yes

Are the datasets clearly presented in a useable and accessible format?

Yes

Reviewer Expertise:

Medical Imaging Technology, MRI

I confirm that I have read this submission and believe that I have an appropriate level of expertise to confirm that it is of an acceptable scientific standard.
